# Image analysis method for measurement and prediction of intra‐matrix IgG diffusion

**DOI:** 10.1002/btpr.70085

**Published:** 2025-11-17

**Authors:** Riya Debbarma, Antonio C. F. dos Santos, Michael Ladisch

**Affiliations:** ^1^ Laboratory of Renewable Resources Engineering Purdue University West Lafayette Indiana USA; ^2^ Department of Agricultural and Biological Engineering Purdue University West Lafayette Indiana USA; ^3^ Engineering Department Morissey College of Arts and Sciences, Boston College Chestnut Hill Massachusetts USA; ^4^ Weldon School of Biomedical Engineering Purdue University West Lafayette Indiana USA

**Keywords:** diffusion, hyaluronic acid (HA) matrix, image analysis

## Abstract

Measurement and imaging of intra‐matrix protein therapeutics diffusion is important due to the emergence of injectable biologics currently in various stages of research and clinical testing. These therapeutics are developed for delivery to hyaluronic acid (HA)‐rich anatomical sites such as subcutaneous tissue, the vitreous humor, and knee joints, depending on the target tissue. Understanding their diffusion behavior is essential for optimizing drug delivery strategies. Our work presents an image analysis method suited for tracking IgG diffusion in low viscosity HA matrices representative of the vitreous humor, where diffusion occurs more rapidly unlike a previously reported analysis method for higher viscosity matrices where protein diffusion is significantly slower. The current method utilizes scanner images at 6.3 MP resolution, and an algorithm that removes background and calculates protein mass and concentration measured directly within matrices formulated to represent HA in an intravitreal environment. We report and demonstrate a robust method for predicting protein diffusion coefficient from images of label‐free protein diffusing in a low viscosity HA matrix.

## Graphical abstract

Experimental and image analysis workflow for b‐IgG diffusion in hyaluronic acid (HA) matrix.

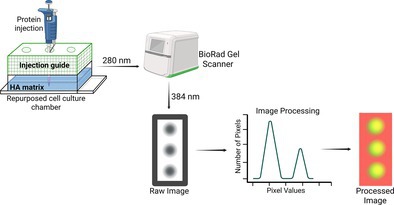

## 1 INTRODUCTION

Major drug administration routes such as subcutaneous (SQ), intravitreal, and intra‐articular are actively being explored to enhance systemic drug bioavailability after site‐specific administration.^1^, ^2^ Among these, SQ delivery remains the most convenient and preferred route, with several novel in vitro tools developed to study antibody transport in a simulated SQ environment.^3^, ^4^ Ultimately the choice of administration route depends on effective delivery to the target tissues and ensuring a high antibody concentration at the target site to improve therapeutic response.^5^

With the aging population, ocular diseases such as age‐related macular degeneration (AMD) and Diabetic retinopathy (DR) are becoming more prevalent, driving the continuous development of intravitreal therapeutics injected directly into the vitreous humor of the eye.^6^, ^7^, ^8^ AMD is expected to increase to ~17.8 million by 2050,^9^ while DR is expected to affect 16 million by 2050.^10^ Several intravitreal drugs that have received FDA‐approval include Ranibizumab (Lucentis®), Aflibercept (Eylea®), and Bevacizumab (Avastin®; off‐label application), with others, such as anti‐IL‐6 antibody (Roche), currently in various stages of development and clinical trial. Intravitreal injection of monoclonal antibodies (mAbs) ensures peak intraocular drug concentration, with transport governed by diffusion.^11^, ^12^

The rate of diffusion of mAbs depends on their molecular weight, hydrophobic character and the viscosity of the formulation.^4^ Therefore, understanding the systemic diffusion of mAbs post‐injection is important for informing formulation, dosing strategies, and designing controlled‐release systems. Developing a reliable in vitro platform is essential for accurate preclinical prediction of mAb diffusion.

Various anatomical locations in the body are rich in hyaluronic acid (HA), with concentrations reported at 500 μg/g of wet tissue weight in the SQ, 2–3 mg/mL in the articular joint synovial fluid, and 200 μg/mL in the vitreous body of the eye.^13^ The vitreous humor is an avascular and nearly acellular compartment,^14^ where HA imparts the anionic nature and viscosity to the vitreous.^12^, ^15^

An in vitro method was developed to simulate SQ drug diffusion using a viscous HA matrix (at 10 mg/mL; 1.5 MDa) that represents the electrostatic environment of the SQ. This method enabled dynamic measurement of unlabeled protein mass in the matrix, facilitated by a complementary method that allows direct calculation of concentration‐dependent diffusion from images of protein movement in an HA matrix.^4^, ^16^

Our work leverages the previously developed in‐vitro platform^4^ and introduces key modifications to simulate IgG antibody transport in the human vitreous. To quantify the mass and concentration of the label‐free protein in our setup, we developed a facile image analysis method as the faster diffusion observed in our HA matrix rendered the earlier method unsuitable, which was developed for a slower diffusion rate. To accommodate the rapid diffusion observed in our experiments, we adapted the imaging protocol to capture the protein diffusion effectively. This also required correcting for the inherent fluorescence of the HA matrix and implementing custom mass standard curves. As a result, we report an efficient and optimized image analysis method well‐suited for tracking label‐free protein diffusion in low viscosity HA matrices.

## 2 MATERIALS AND METHODS

### 2.1 Preparation of hyaluronic acid (HA) matrices and injection protein

Sodium hyaluronate powder (Lot #030386, 0.5 MDa; Lifecore Biomedical Chaska, MN) 200 and 400 mg was dissolved in 20 mL of phosphate buffered saline (PBS, pH 7.4; Sigma Aldrich, St. Louis, MO) in a borosilicate round bottom centrifuge tube (DWK Life Sciences) to obtain a final concentration of 10 and 20 mg/mL, respectively. The solutions were mixed overnight using a rotisserie tube rotator (Scilogex, Rocky Hill, CT) and transferred to a 4°C refrigerator for 2–4 h for degassing.^4^

Bovine gamma globulin (Bovine IgG) lyophilized powder (Lot #SLBZ8713; Sigma‐Aldrich, St. Louis, MO) was used to prepare the injection protein. Thousand milligrams of bovine IgG (b‐IgG) was dissolved in 10 mL PBS with 0.05% Tween®80 (Sigma‐Aldrich, St. Louis, MO) and mixed overnight at room temperature using a rotisserie tube rotator (Scilogex, Rocky Hill, CT). The bovine IgG stock solution was diluted to 50 mg/mL for injection into the HA matrix. The concentration was measured using 280 nm absorbance in Take 3 nanoplates in an Epoch 2 microplate spectrophotometer (Biotek US, Winooski, VT).

### 2.2 Imaging protocol

**FIGURE 1:**
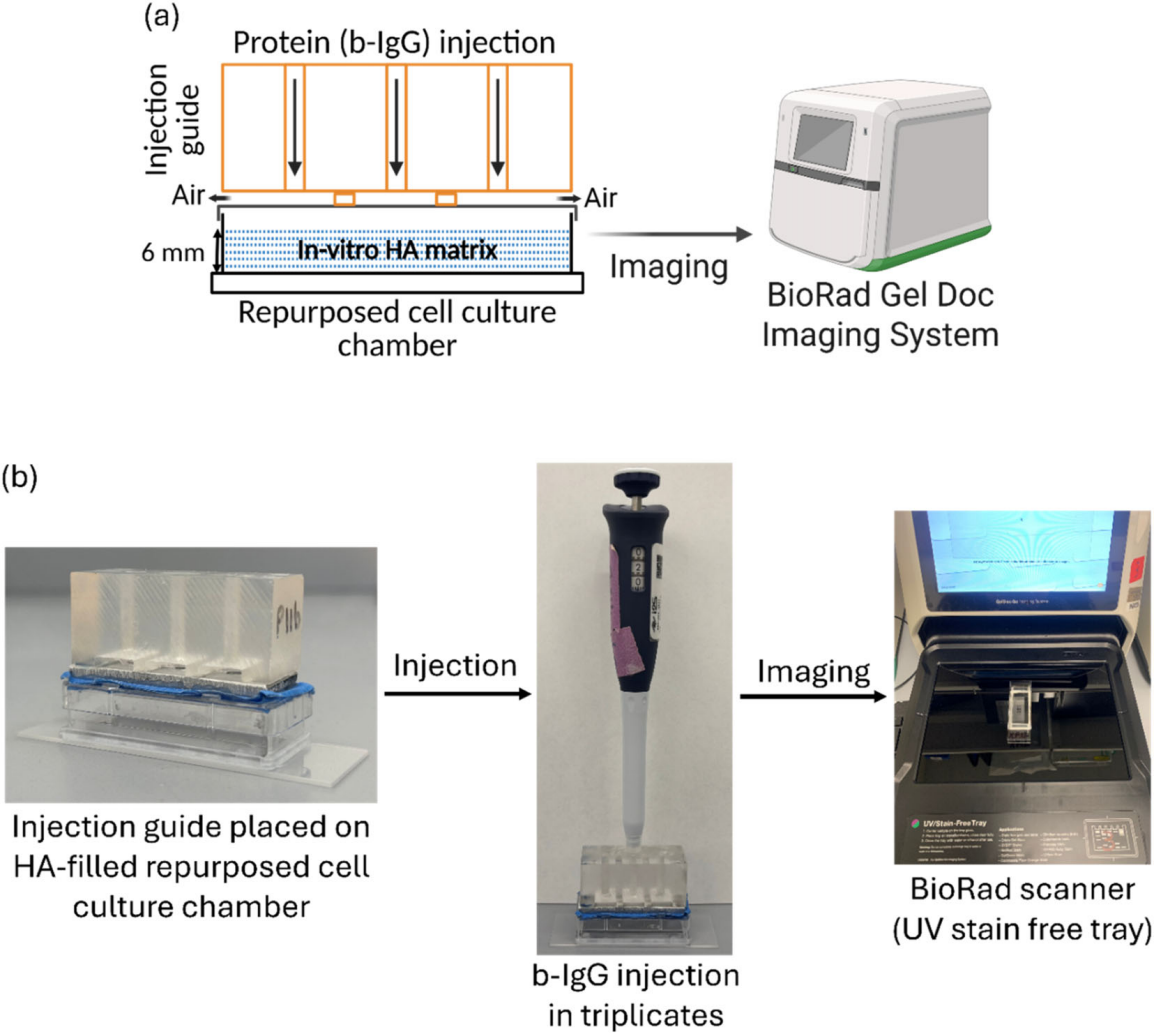
Diffusion experiment setup (a) Graphical representation (Created in BioRender. Debbarma, R. (2025) https://BioRender.com/rk61ur5) (Drawing of repurposed cell culture chamber reproduced from the graphical abstract of the manuscript accepted by Molecular Pharmaceutics, October 30, 2025)^17^ (b) Illustration of the components utilized to make the measurements.

Imaging of b‐IgG diffusion in the HA matrix was done label‐free using our previously reported method of activating the tryptophan residues at 280 nm and capturing the protein autofluorescence at 384 nm; 6.6 mL of the HA solution was pipetted into a repurposed cell culture chamber to achieve a depth of 6 mm; 20 μL of b‐IgG (50 mg/mL) was injected into the HA matrix in triplicates and its movement was imaged using a gel imaging scanner^4^ (BioRad Gel Doc Imaging Systems with a UV stain‐free tray) (Figure 1).

Activation and diffusion imaging times were adjusted based on HA concentration: for 10 mg/mL HA, activation was performed for 45 s and diffusion was monitored every 1 min for 3–5 min; for the 20 mg/mL HA, activation was extended to 5 min with diffusion imaged every 5 min for 15–20 min. The diffusion experiments were conducted over three experimental days, with two replicates per day. Of the six total replicates, one was used to demonstrate the step‐by‐step image processing method in this paper, while the other similar images were used in an accepted manuscript to illustrate the image processing workflow.^17^ The step‐by‐step logic for image processing has been shown in detail with examples in the Supporting Information.

### 2.3 Protein standard curves

**FIGURE 2:**
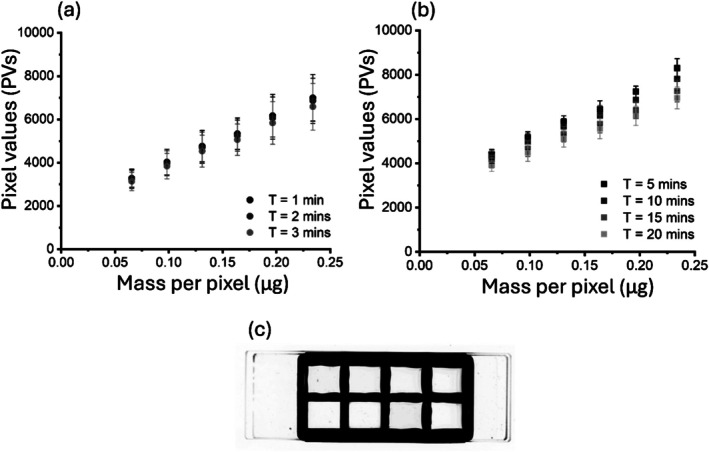
Matrix‐specific protein standard curves for diffusion image analysis in (a) 10 mg/mL HA and (b) 20 mg/mL HA. (c) Eight‐well device for obtaining the standard curves.

Two independent 2 mL protein standard solutions were prepared by diluting the b‐IgG stock solution to 14, 21, 28, 35, 42, and 50 mg/mL, and adding 6.7 and 13.34 mg of HA powder to represent the injected bovine IgG in 10 and 20 mg/mL HA matrices, respectively. The solutions were mixed overnight using a rotisserie tube rotator and refrigerated at 4°C for degassing. The standard curve was obtained by pipetting 200 μL b‐IgG + HA standard solutions into the eight‐well cell and imaging under the same matrix‐specific conditions as described in Section 2.2 (Figure 2c). The images were processed using a previously described method.^4^, ^16^ Due to meniscus formation, the true depth of the standard solutions in the eight‐well cell was 1 mm, which was used for calculating the pixel volume and mass per pixel value of the standards (Equations 1 and 2). At each timepoint, the slope and intercept of the standard curve were obtained by plotting mass per pixel versus pixel values (Figure 2a,b) (Tables 1 and 2).

**TABLE 1:** Slope and intercept from protein standard curve for diffusion image analysis in 10 mg/mL HA.

Timepoint (min)	Slope	Intercept
1	21890.59 ± 3869.24	1850.78 ± 195.61
2	21348.85 ± 3722.59	1846.69 ± 203.69
3	20324.40 ± 3816.44	1818.17 ± 207.41

**TABLE 2:** Slope and intercept from protein standard curve for diffusion image analysis in 20 mg/mL HA.

Timepoint (min)	Slope	Intercept
5	22467.70 ± 1118.94	2908.33 ± 157.68
10	20326.68 ± 1231.76	2951.82 ± 231.20
15	18549.76 ± 1258.15	2836.95 ± 232.97
20	17552.93 ± 1356.82	2764.57 ± 286.73

Pixel size=68.4μm (as registered by the CMOS)

(1) $$\begin{array}{c} \text{Pixel volume}=\text{Pixel area}\times \text{Pixel depth}\\ ={\left(0.00684\right)}^{2}\times 0.1\\ =4.68\times 10^{-6}\;{\mathrm{cm}}^{3} \end{array}$$

(2) $$\text{Mass}\;\mathrm{per}\;\text{pixel}\left(\mathrm{\mu g}\right)=\begin{array}{l} \text{Protein standard concentration}\left(\mathrm{mg}/\mathrm{mL}\right)\\ \times \text{Pixel volume}\times 1000 \end{array}$$

### 2.4 Diffusion image pixel value extraction and processing

**FIGURE 3:**
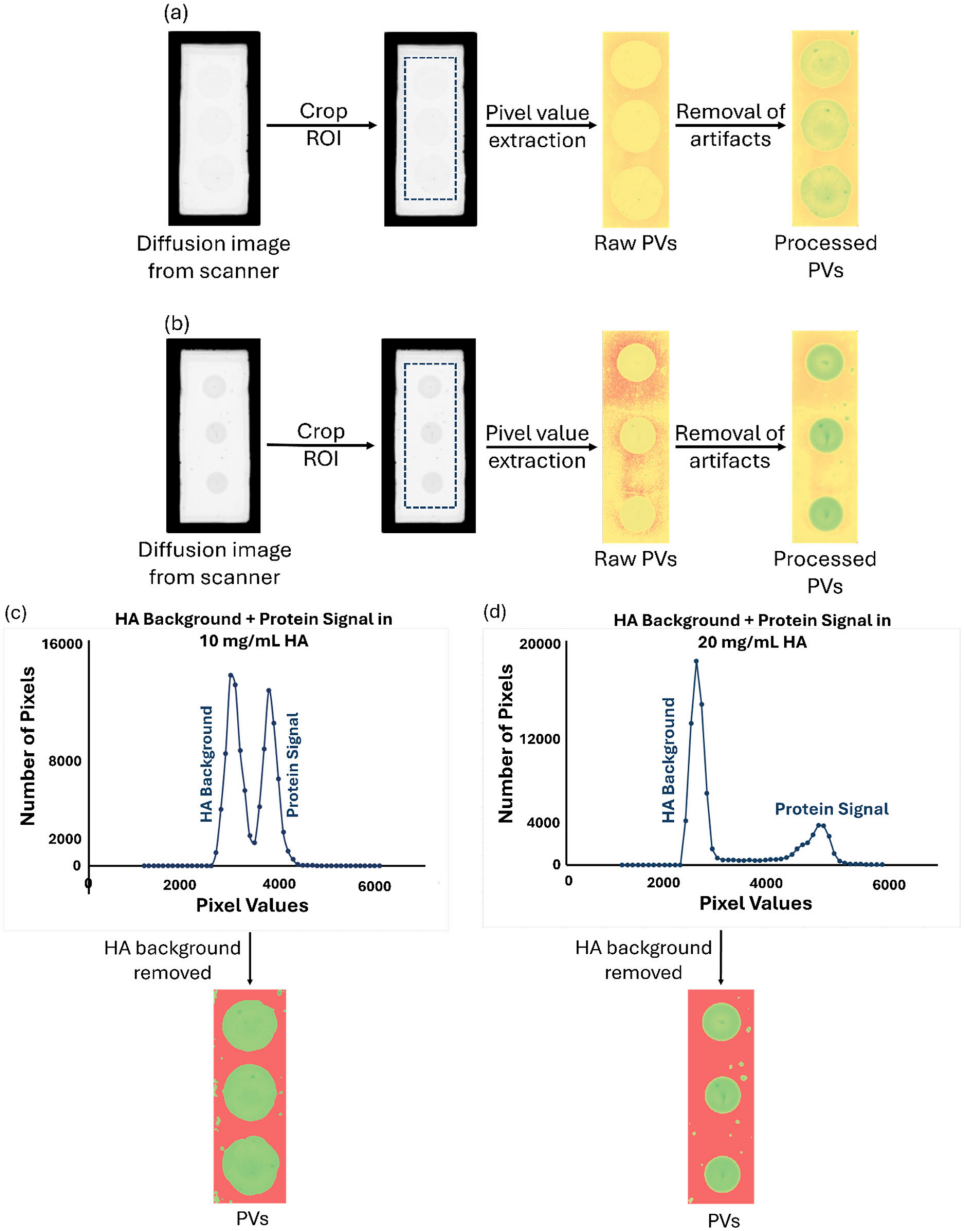
Pixel value extraction and processing from b‐IgG diffusion images. (a) At 1 min timepoint in 10 mg/mL HA (b) At 5 min timepoint in 20 mg/mL HA. (c, d) Background detection and removal for images in (a) and (b), respectively.

The b‐IgG diffusion images captured by the complementary metal‐oxide‐semiconductor (CMOS) sensor in the BioRad gel scanner were processed and converted to pixel values using MATLAB R2022b (MathWorks). Images (TIF format) acquired at each timepoint were vertically aligned and cropped to the region of interest (ROI) as shown by a dotted rectangle in Figure 3a,b.^16^, ^18^ Each image consisted of ~75,000 pixels, with pixel values (PVs) representing the light intensity. Outlier PVs from artifacts such as dust specks were removed using a 5 × 5 moving median filter, followed by 2D Gaussian smoothing (σ = 2 pixels) (Figure 3a,b).

### 2.5 Background detection and removal

To determine the background in the cropped diffusion images, a histogram of the pixel values (PVs) versus pixel count was generated using a bin width of 100. The PV with the highest pixel count (frequency), corresponding to the first peak in the histogram, was identified as the background of the image.^19^ PVs ≤ background PV was set to zero, while the remaining non‐zero PVs represented the protein (b‐IgG) signal (greenish yellow boluses).^16^, ^18^ For accurate protein mass estimation from PVs, it is important to evaluate the histogram and identify whether the background and the protein signal form well‐separated peaks or overlapping but distinguishable peaks (Figure 3c,d). This has been discussed in detail in Section 3.3.

### 2.6 Pixel to mass conversion and HA fluorescence correction

**FIGURE 4:**
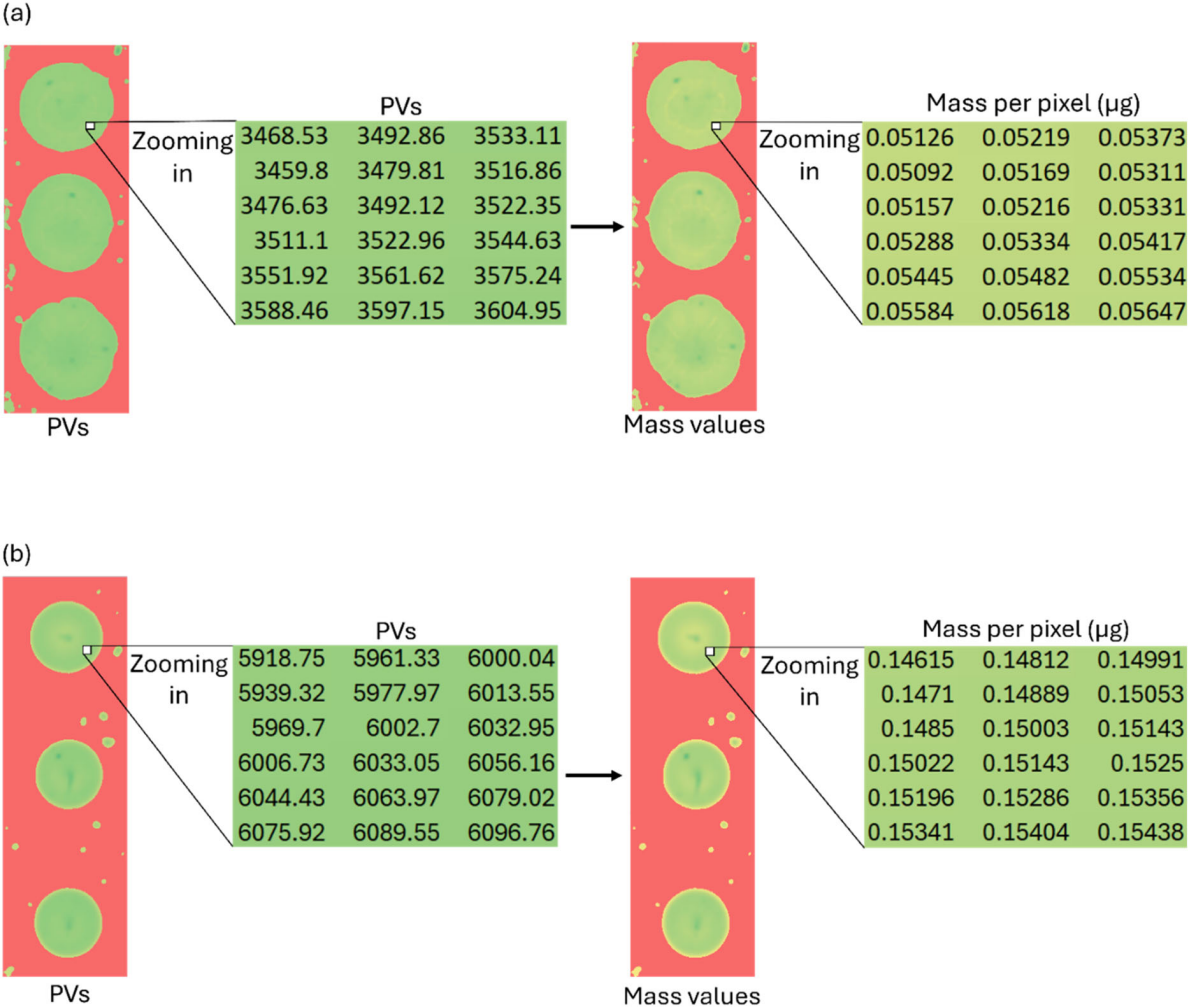
b‐IgG pixel values converted to mass using matrix‐specific protein standard curves in (a) 10 mg/mL HA at the 1 min timepoint and (b) 20 mg/mL HA at the 5 min timepoint.

The non‐zero PVs (protein signal) were converted to protein mass per pixel using the b‐IgG + HA standard curve. At each timepoint, protein mass per pixel was estimated using the slope and intercept from the standard curve as described in Section 2.3 (Figure 4a,b) (Equation 3).

(3) $$\text{Mass}\;\mathrm{per}\;\text{pixel of injected}\;b-\mathrm{IgG}\;\left(\mathrm{\mu g}\right)=\frac{\text{Non}-\text{zero}\;\mathrm{PV}-\text{Intercept}}{\text{Slope}}$$

For b‐IgG diffusion in 20 mg/mL HA, the two histogram peaks are well‐separated, so background correction is not necessary (Figure 3d). In contrast, diffusion in 10 mg/mL HA shows two overlapping yet distinguishable peaks (Figure 3c), hence the mass contribution from HA fluorescence must be subtracted to avoid overestimating the protein mass.

**FIGURE 5:**
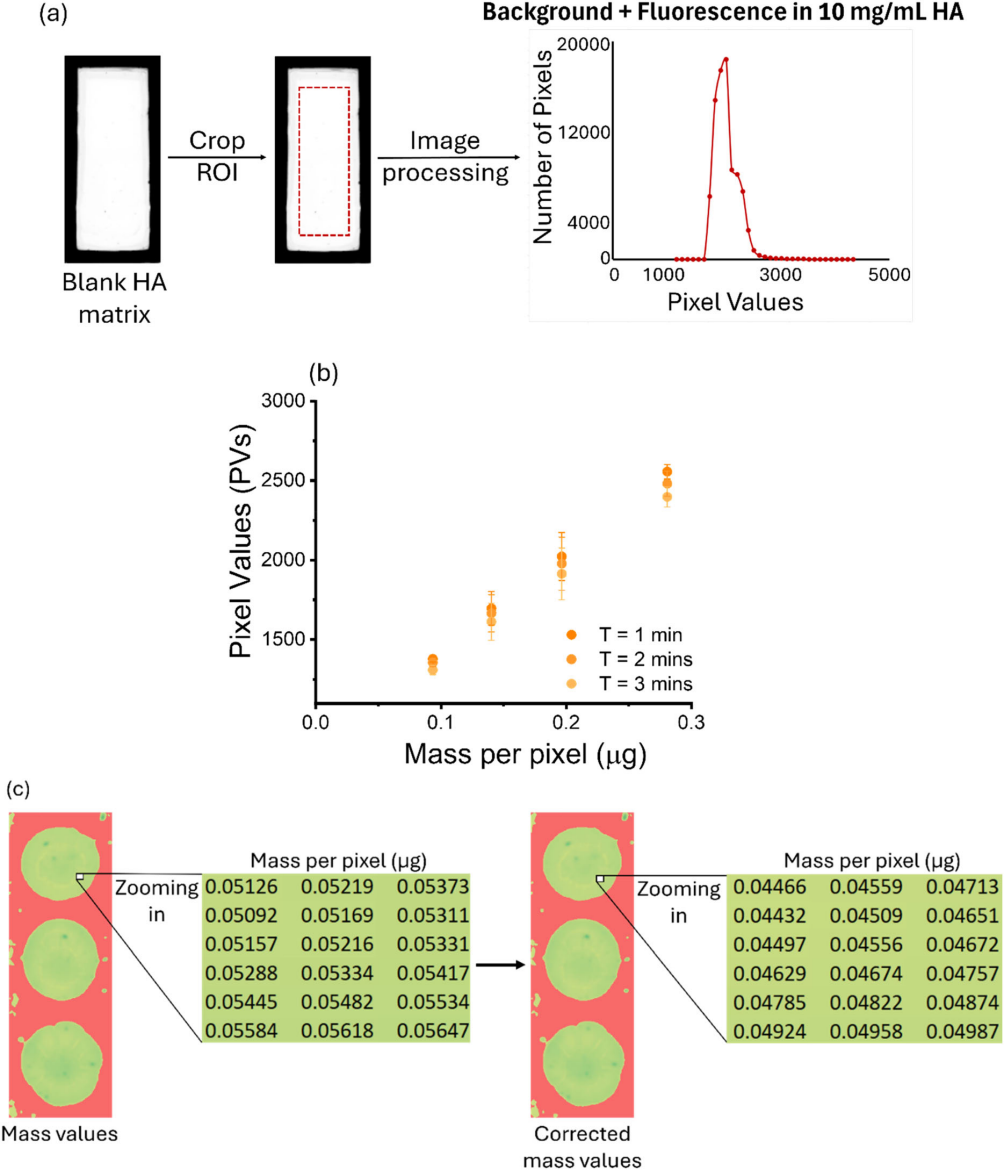
HA fluorescence correction using (a) a blank HA matrix and (b) an HA‐only standard curve. (c) HA fluorescence subtraction shown for the image in Figure 4a.

To account for this, a blank HA matrix was imaged and processed using conditions identical to the diffusion images (Figure 5a), and the HA fluorescence values were obtained using the HA‐only standard curve. These curves were generated by pipetting 600 μL of 3.33, 5, 7, and 10 mg/mL HA into an eight‐well cell (6 mm depth), followed by imaging and processing under the same conditions as the b‐IgG + HA standard curves (Figure 5b). At each timepoint, the slope and intercept from the HA‐only standard curve (Table 3) were used to estimate the mass values that account for HA fluorescence (~0.002–0.013 μg), which were then subtracted from the mass per pixel values obtained from the diffusion images (Figure 5c).

**TABLE 3:** Slope and intercept from HA‐only standard curve to obtain fluorescence value of 10 mg/mL HA.

Timepoint (min)	Slope	Intercept
1	6235.12 ± 97.76	805.32 ± 63.16
2	5946.39 ± 252.67	813.05 ± 52.24
3	5745.97 ± 138.88	787.37 ± 68.63

### 2.7 Mass recovery and validation

The accuracy of the calculated protein mass per pixel was validated by summing the mass across all pixels and comparing it to the known injected protein load, ensuring that for every replicate, the estimated total mass fell within ±20% of the injected mass.

In our experiments, the injected protein load was ~0.94–1.04 mg. The ±20% mass recovery range was selected based on experimental observations and the 95% confidence interval (CI) of the mean recovered mass from the diffusion images. This range accounts for reduced protein autofluorescence over time, as the signal depends on imaging duration, activation time, and potential instrumental drift. It also accommodates the slight overestimation by the b‐IgG + HA standard curve as the injected protein gradually dissolves into the HA matrix over time.

### 2.8 Mass to concentration conversion

**FIGURE 6:**
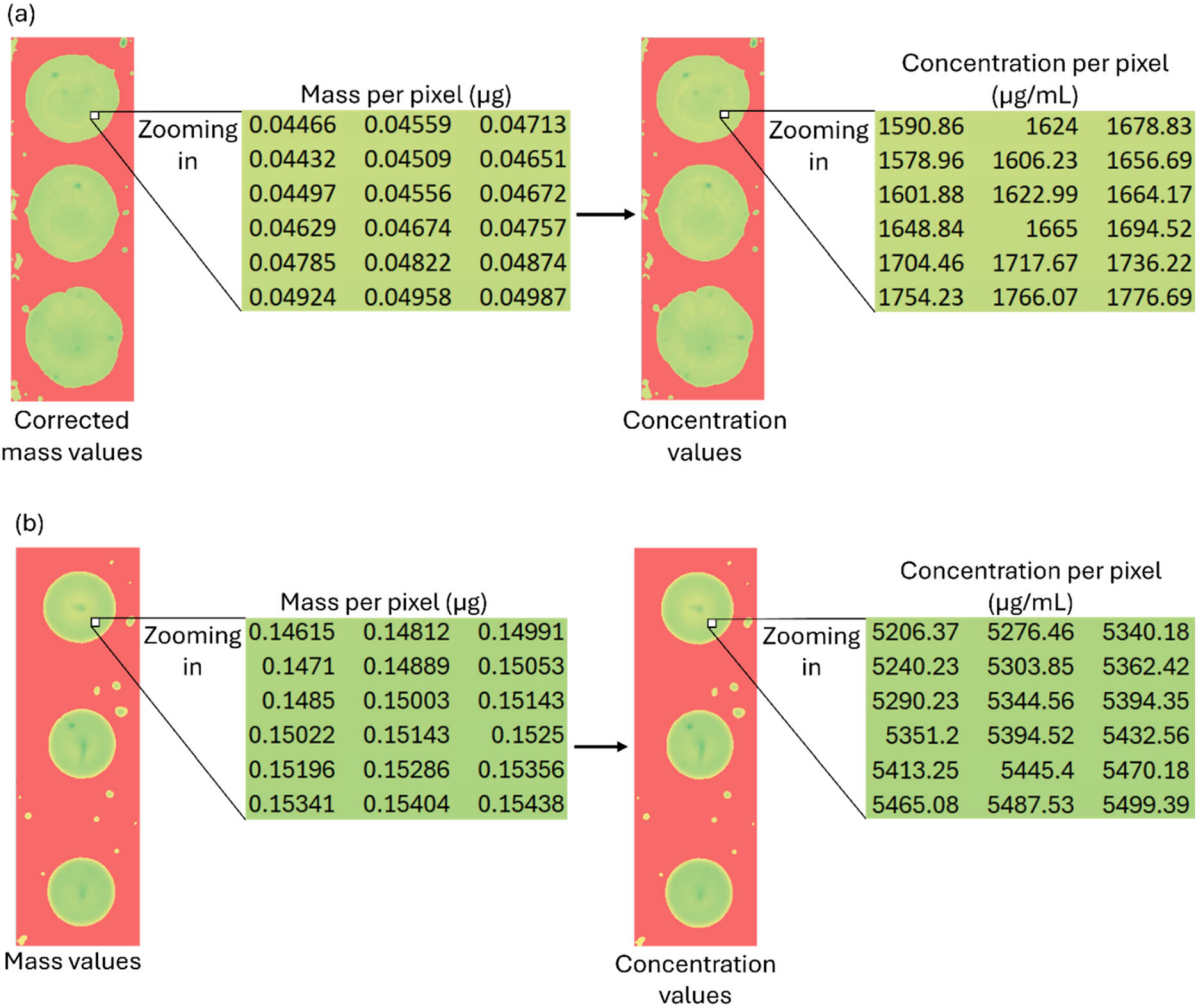
b‐IgG mass values converted to concentration using pixel volume in (a) 10 mg/mL HA at the 1 min timepoint and (b) 20 mg/mL HA at the 5 min timepoint.

The b‐IgG mass per pixel values were converted to concentration per pixel using the pixel volume, which was determined based on the known pixel size as registered by the CMOS at a given pixel depth (Equations 4 and 5) (Figure 6a,b).

(4) $$\begin{array}{c} \text{Pixel volume}=\text{Pixel area}\times \text{Pixel depth}\\ ={\left(0.00684\right)}^{2}\times 0.6\;\\ =2.81\times 10^{-5}\;{\mathrm{cm}}^{3} \end{array}$$

(5) b−IgGconcentrationperpixelμg/mL=b−IgGmassperpixelPixel volume

## 3 RESULTS AND DISCUSSION

### 3.1 Prediction of effective diffusion coefficients

The median b‐IgG concentration was calculated from the local protein concentrations across all pixel values. For each of the triplicate injections, the median concentration was used to determine the effective diffusion coefficient (D_eff_ in cm^2^/s) based on the fitted function for concentration‐dependent diffusion of b‐IgG in HA matrix^18^ (Equation 6).

(6) $$D_{\mathrm{eff}}=2.993\times 10^{-7}\exp\left(-0.199\left[\mathrm{IgG}\right]\right)$$

where [IgG] is the median concentration of the injected b‐IgG in mg/mL.

### 3.2 Analysis of the b‐IgG median concentration and D_eff_

**FIGURE 7:**
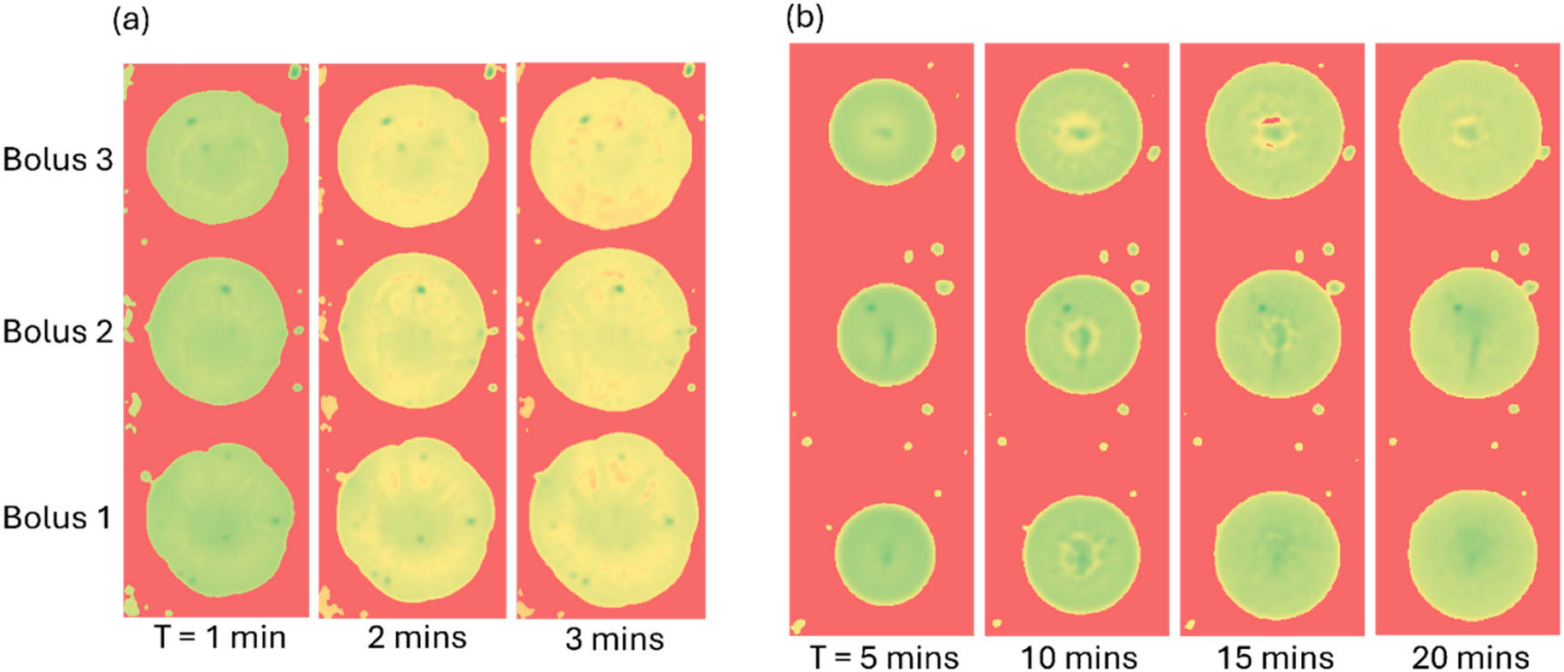
Processed images showing the concentration gradient resulting from b‐IgG diffusion over time in (a) 10 mg/mL HA and (b) 20 mg/mL HA.

The median concentrations and corresponding predicted D_eff_ are reported in Tables 4 and 5. Analysis of each bolus over time (Figure 7a,b), clearly reveals a concentration gradient as b‐IgG diffuses in the HA matrix. This gradient is reflected in the decreasing median concentration reported in Tables 4 and 5. It has been noted that the instantaneous diffusion coefficient changes over time as diffusion progresses due to decreasing concentration.^18^ Consistently, our results showed that as diffusion proceeds, the median concentration of the b‐IgG bolus decreases, leading to an increase in D_eff_.

**TABLE 4:** D_eff_, median concentration and mass recovery for b‐IgG diffusion in 10 mg/mL HA.

Bolus number	Time (min)	Median concentration (mg/mL)	Diffusion coefficient (D_eff_) × 10^−7^ (cm^2^/s)	Mass recovery (mg)
Bolus 1	1	1.78 ± 0.09	2.10 ± 0.04	1.03 ± 0.06
	2	1.64 ± 0.07	2.16 ± 0.03	1.11 ± 0.08
	3	1.54 ± 0.05	2.20 ± 0.02	1.18 ± 0.13
Bolus 2	1	1.66 ± 0.09	2.15 ± 0.04	0.95 ± 0.06
	2	1.52 ± 0.11	2.21 ± 0.05	1.01 ± 0.09
	3	1.42 ± 0.05	2.26 ± 0.02	1.08 ± 0.13
Bolus 3	1	1.58 ± 0.18	2.19 ± 0.08	0.89 ± 0.10
	2	1.46 ± 0.09	2.24 ± 0.04	0.96 ± 0.12
	3	1.36 ± 0.12	2.29 ± 0.05	1.02 ± 0.20

**TABLE 5:** D_eff_, median concentration and mass recovery for b‐IgG diffusion in 20 mg/mL HA.

Bolus number	Time (min)	Median concentration (mg/mL)	Diffusion coefficient (D_eff_) × 10^−7^ (cm^2^/s)	Mass recovery (mg)
Bolus 1	5	4.07 ± 0.27	1.33 ± 0.07	0.81 ± 0.08
	10	3.29 ± 0.41	1.56 ± 0.13	0.85 ± 0.10
	15	3.06 ± 0.39	1.63 ± 0.13	0.89 ± 0.12
	20	2.79 ± 0.39	1.72 ± 0.13	0.90 ± 0.15
Bolus 2	5	3.94 ± 0.31	1.37 ± 0.08	0.87 ± 0.17
	10	3.43 ± 0.42	1.52 ± 0.13	0.91 ± 0.18
	15	3.11 ± 0.50	1.62 ± 0.16	0.95 ± 0.21
	20	2.80 ± 0.53	1.72 ± 0.19	0.96 ± 0.20
Bolus 3	5	3.57 ± 0.45	1.48 ± 0.13	0.81 ± 0.11
	10	3.00 ± 0.48	1.65 ± 0.16	0.82 ± 0.11
	15	2.70 ± 0.51	1.75 ± 0.18	0.84 ± 0.14
	20	2.47 ± 0.52	1.84 ± 0.19	0.84 ± 0.14

Statistical analysis (paired *t*‐test) showed a significant difference in D_eff_ between the boluses due to injection time difference, and Welch's *t*‐test revealed a highly significant difference in D_eff_ values between matrices. These findings have been discussed in detail in an accepted manuscript.^17^ Furthermore, the accuracy of D_eff_ estimation was confirmed by the experimentally obtained total b‐IgG mass recovery of 0.80–1.30 mg in 10 mg/mL HA and 0.70–1.18 mg in 20 mg/mL HA, which fell within the expected range. Day‐to‐day variability in D_eff_ values was within 4% for the 10 mg/mL HA matrix and 11% for the 20 mg/mL HA matrix, demonstrating experimental reproducibility.

### 3.3 Key considerations for imaging protocol

In our experiments, b‐IgG diffusion was measured in 10 mg/mL and 20 mg/mL HA matrices. It has been observed that the diffusion rates of globular proteins such as bovine serum albumin and human γ‐globulin decrease with increasing HA concentrations,^20^ highlighting the importance of setting the tryptophan activation time and diffusion imaging protocol accordingly. Imaging the diffusion of unlabeled b‐IgG in the HA matrix requires UV activation of the tryptophan residues to enable visualization of b‐IgG against the HA background. Activation time in the gel scanner was selected based on experimental observations. In our preliminary experiments, b‐IgG reached the chamber walls within 3 min of injection in the 10 mg/mL HA, compared to 30 min in the 20 mg/mL HA. The BioRad gel scanner used for imaging offers only two preset activation time settings: 45 s and 5 min. For accurate measurement of diffusion, activation of the protein must be done before it reaches the chamber walls, while also ensuring that the protein signal is maintained throughout the entire diffusion period. Accordingly, a 45‐s activation time was used to image b‐IgG diffusion in the 10 mg/mL HA, while the 5‐min setting was used in the 20 mg/mL HA. However, in the 10 mg/mL HA, the shorter activation time prevents the development of a distinct protein signal, resulting in an overlap between the HA background and protein signal peaks (Figure 3c). Therefore, the two peaks must be deconvoluted by subtracting the HA fluorescence (Figure 5), as described in Section 2.6. In the 20 mg/mL HA, longer activation time improved the contrast between HA matrix background and protein signal, resulting in well‐separated histogram peaks and eliminating the need for HA fluorescence correction (Figure 3d). Lastly, matrix‐specific b‐IgG + HA standard curves were essential to accurately estimate the injected protein mass from the pixel values. The depth of the b‐IgG + HA and HA‐only standard curves must be selected to closely match the diffusion experiment setup while ensuring signals remain within the detectable range and avoid oversaturation, particularly for the b‐IgG + HA standards.

### 3.4 Practical considerations and limitations

**FIGURE 8:**
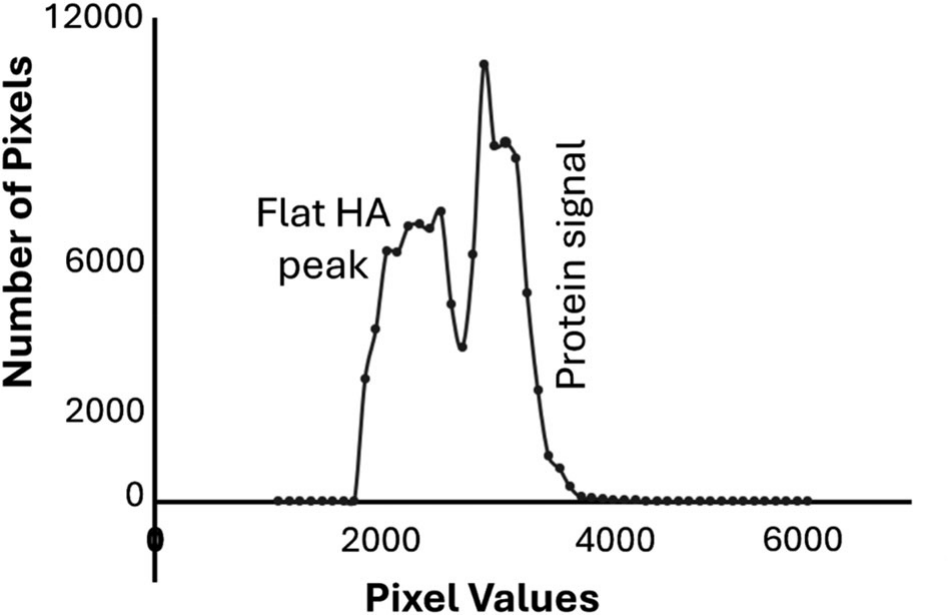
Flattening of HA peak due to repeated UV exposure of the polycarbonate chamber used in diffusion experiments.

Repeated UV exposure in the BioRad scanner causes the polycarbonate cell culture chamber to lose its transparency, subsequently casting a shadow on the HA matrix. This leads to an increase in the background of the HA matrix, thereby interfering with the protein signal. This was observed in one of the replicates where the HA peak flattened and interfered with the protein signal hindering the HA background detection due to which the data were discarded from further analysis. This determines the longevity of the current chamber and indicates the need for periodic replacement (Figure 8). A newly assembled chamber can be used continuously for months, although it is recommended to replace the cell after 10–14 h of use. Currently, only the bottom of our chamber is a quartz slide, making the base UV transparent, while the rest is polycarbonate. A more durable alternative would be to construct the entire chamber from quartz, which would eliminate the need for frequent replacements and allow for extended, potentially indefinite, use. As for the biological relevance of our in vitro platform, previous studies have reported that vitreous liquefaction begins after the age of 40, and by 80–90 years of age, more than 50% of the vitreous is liquid.^21^ This process is characterized by phase separation, resulting in pockets of liquid rich in HA.^22^ Therefore, the in vitro HA matrices closely mimic the vitreous of an aging population.

### 3.5 Limitations of the method for determining D_eff_

Our method for measuring b‐IgG D_eff_ in HA matrices relies on ensemble‐average measurements and assumes Fickian diffusion. However, diffusion in complex polymeric environments can deviate from this ideal behavior. Previous work using fluorescence correlation spectroscopy has shown that even in dilute polymer solutions, complex tracer diffusion can be observed due to specific tracer‐polymer interactions, with a second diffusion component dependent on polymer chain length.^23^ In addition, polymer solutions exhibit large length‐scale heterogeneity, and traditional ensemble average experiments may not capture anomalous diffusion.^24^, ^25^ Therefore, complementary spectroscopic methods that focus on interfaces and single‐molecule dynamics may be needed in addition to our method to measure anomalous diffusion.

## 4 CONCLUSION

We present an intuitive and user‐friendly image analysis method for tracking diffusion of unlabeled protein in low viscosity HA matrices, providing an accurate and reproducible quantification of protein mass and concentration. Its accuracy is confirmed by protein mass recovery within ±20% of the injected load following image analysis. The method lays groundwork for further optimization and adaptation to monitor drug diffusion in customized hydrogels that mimic HA and collagen‐rich anatomic locations in the body. While ensemble experiments are useful for identifying the type of interactions that may dominate protein transport, fundamental studies using complementary techniques such as FRAP, single‐particle tracking, and modeling of tracer dynamics in various hydrous polymeric environments are needed for detailed mechanistic insights. The approach can also be extended to tracking nanoparticle diffusion, offering broad application in drug delivery studies. Additional improvements are underway to expand the applicability of the method.

## AUTHOR CONTRIBUTIONS

**Riya Debbarma:** Writing‐review and editing; design and carry out experiments; experimental methodology; interpretation of data and results; analysis; visualization. **Antonio C. F. dos Santos:** Reviewing; interpretation of results; analysis. **Michael R. Ladisch:** Conceptualization and mechanisms; design of experiments; interpretation of data and results; writing‐review and editing; funding.

## FUNDING INFORMATION

This work was supported by Laboratory of Renewable Resources Engineering, Purdue University; Department of Agricultural and Biological Engineering, Purdue University; and USDA Hatch Project IN90027984.

## CONFLICT OF INTEREST STATEMENT

The authors declare no conflict of interest.

## Supporting information


**Data S1:** Supplementary Information.

**Video S1:** Procedure for image analysis: step‐by‐step explanation and example.

## Data Availability

The data that support the findings of this study are available from the corresponding author upon reasonable request.
