# Supplementary material for: Image analysis method for measurement and prediction of intra‐matrix IgG diffusion
Source: Biotechnol Prog. 2025 Nov 17;42(3):e70085. doi: 10.1002/btpr.70085 (PMC13266960; doi:10.1002/btpr.70085)

**Supplementary Material**

***Step-by-step logic for image processing***

Step 1: Crop Region of Interest (ROI) and Pixel Value Extraction

Define input and output path for the images

Validate input to check if a user-defined number of images are available

Load images and convert it to grayscale if in RGB

Rotate image at ±0.1° increments up to ±5° if needed

Draw user-defined rectangular ROI on the image and record ROI position

For each image, ensure ROI is within image boundaries

Save cropped images to an output folder

Save the pixel values for each image to an excel sheet

Step 2: Removal of artifacts using Moving Median

Define the input path of the excel file containing the pixel values

Get excel sheet names and loop through each sheet

Convert data to numeric format

Apply 5x5 moving median filter

Save the filtered pixel values for each image to an excel sheet

Step 3: Gaussian Smoothing

Define the input path of the excel file containing the filtered pixel values

Define sheet names

Define Gaussian smoothing parameter (e.g., σ = 2)

Loop through each sheet

Convert data to a matrix (assuming numeric data)

Apply Gaussian smoothing

Save the smoothed pixel values for each image to an excel sheet

Step 4: Background Detection

Define the input path of the excel file containing the smoothed pixel values

Define bins for histogram (Bins from 1050 to 6050 with step 100)

Initialize cell array for writing histograms

Loop through each sheet

Flatten the matrix to a single vector (in case it is a 2D image)

Compute histogram and write it to a new excel file

Plot histogram if needed

For each image, use the first peak of the histogram to determine the background threshold value

Step 5: Background removal

Define the input path of the excel file containing the smoothed pixel values

Define threshold values for each sheet (Update these values as needed)

Loop through each sheet and process pixel values

Apply thresholding: Set values <= corresponding threshold to zero

Save the background removed pixel values to an excel sheet

Step 6: Conversion of Pixel Values to Mass Values

Step 6.1: Determine slope and intercept from Protein Standards (Ahmadzadegan et. al., 2022)

Add the required Bio-Formats MATLAB toolbox path

Define input path for the protein standard images from the scanner

Specify the number of wells in each image (8 wells in our case)

Loop over each image folder and for each folder, convert the path string to character format for compatibility.

Process individual wells for every image, apply median filtering and gaussian smoothing.

Generate a matrix file containing a time-series image stack

Load the image matrix; compute and store (in excel) mean, median and standard deviation of pixel intensities across timepoints

Step 6.2: Pixel to Mass

Define slope and intercept for each excel sheet (image)

Define the input path of the excel file containing the background removed pixel values

Get sheet names from the input file and check if the number of sheets matches the expected slopes and intercepts

Loop through each sheet

Compute mass values using the formula: (pixel values - intercept) / slope

Replace negative mass values with zero

Write the mass values to a new sheet in the output excel file

Step 7: Determining HA Fluorescence

Repeat Step 1 to 6 using the image of a blank HA matrix and HA standards

Step 8: Removal of HA Fluorescence

Define the input path of the excel file containing the mass values

Define different HA fluorescence values for each sheet (modify these values as needed)

Get sheet names from the input file and check if the number of sheets matches the expected slopes and intercepts

Loop through each sheet

Subtract the corresponding HA fluorescence values

Replace negative mass values with zero

Write the adjusted mass values to a new sheet in the output excel file

(Note: It has to be decided by the user whether the subtraction of HA fluorescence values is required or not by analyzing the histogram of the processed images. Skip step 7 and 8 if not needed. Details have been discussed in the manuscript)

Step 9: Conversion of Mass Values to Concentration Values

Define the pixel volume (Calculated using pixel size and depth)

Define the input path of the excel file containing the mass values or adjusted mass values

Process and read mass values from each excel sheet

Convert mass values to concentration values using the formula: mass values/pixel volume

Write concentration values to a new sheet in the output excel file

Step 10: (In Excel)

Determine total mass of each injection bolus

Determine median concentration of each injection bolus (3 boluses in our case)

Calculate effective diffusion coefficient (D_eff_) using Equation 6.

***Example***

Images shown here are b-IgG diffusion image in 10 mg/mL HA captured at T = 1 min in one of the scanners for a particular experimental day

PV extraction and processing:


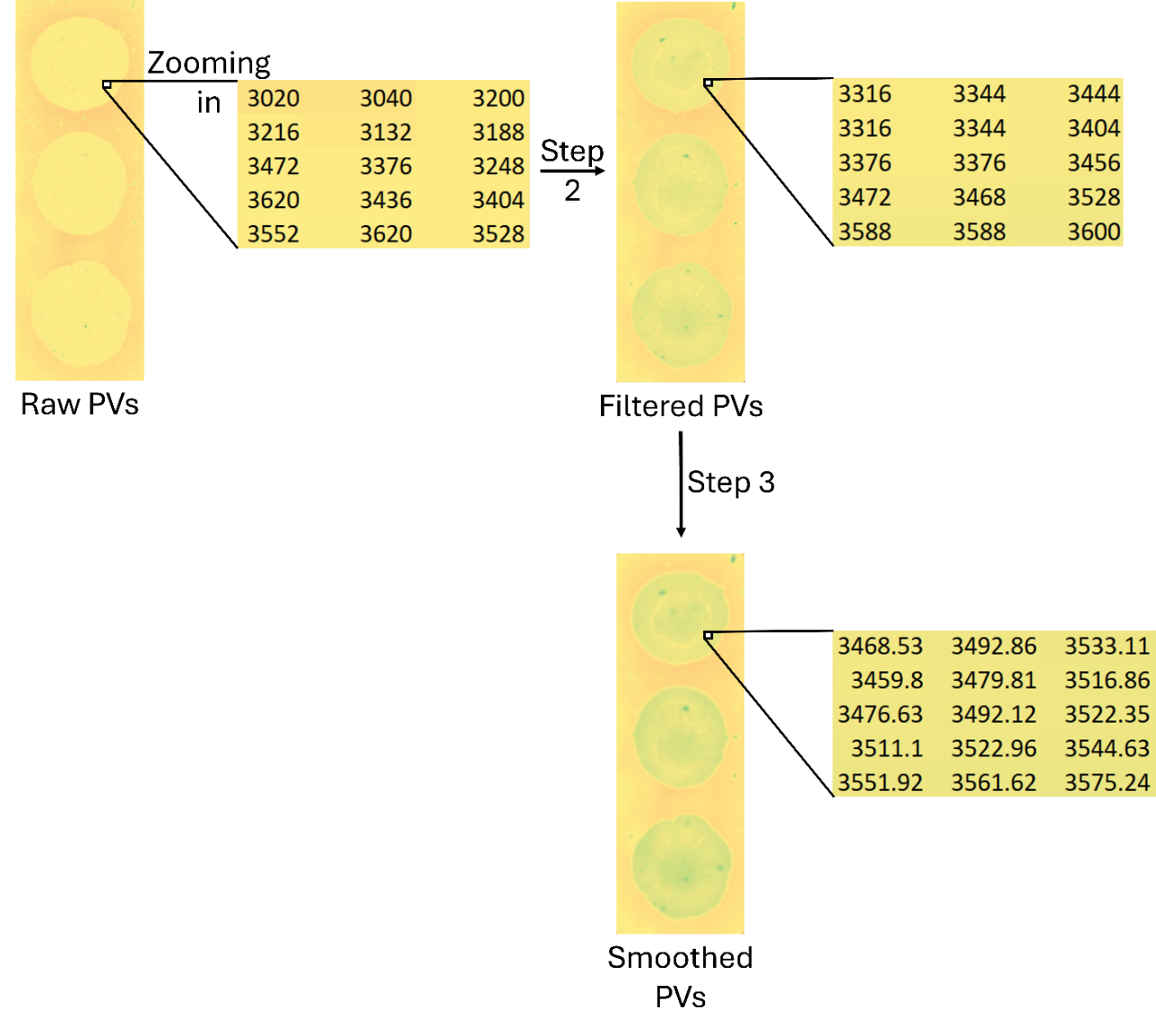


Background removal:


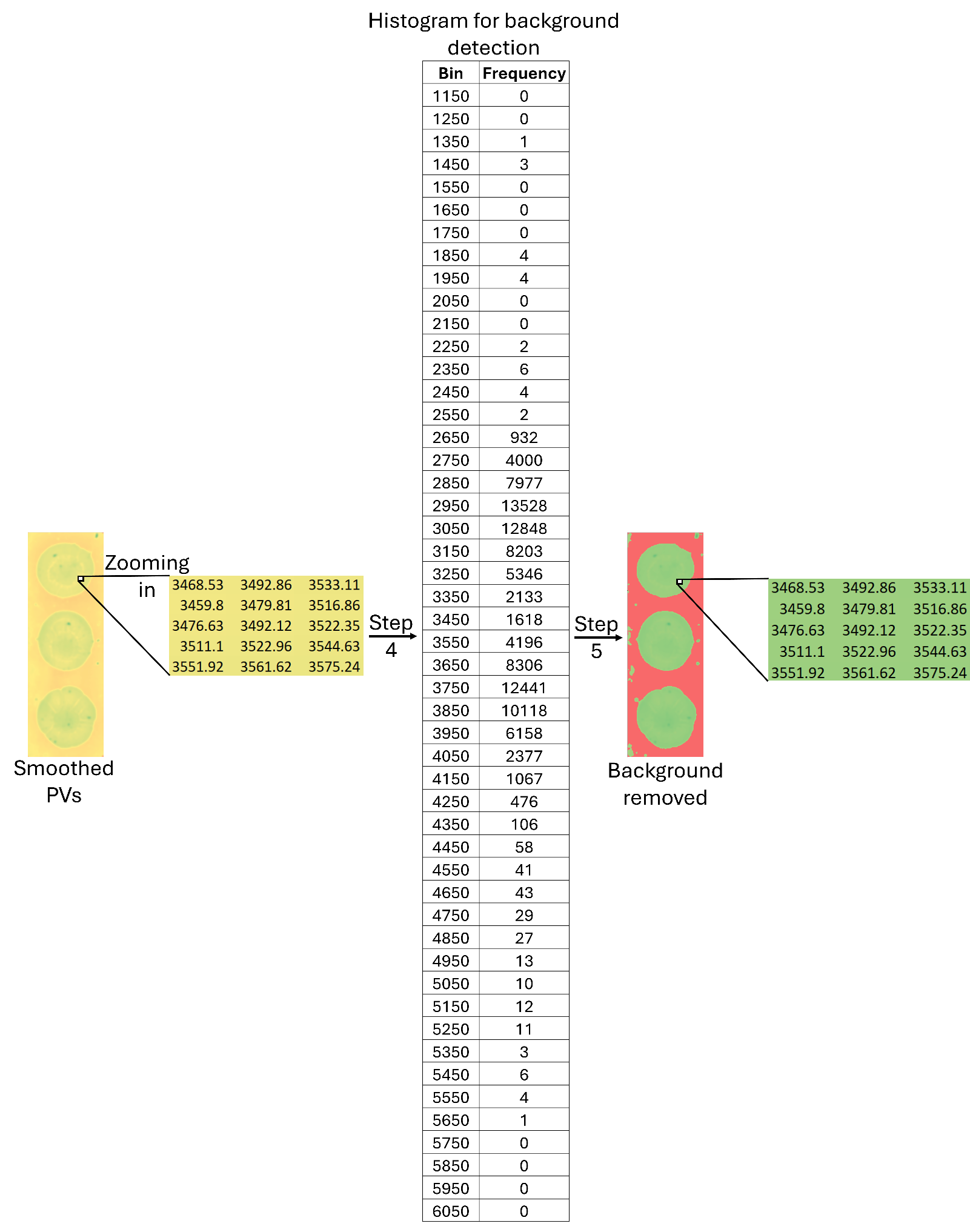


PV to mass conversion:


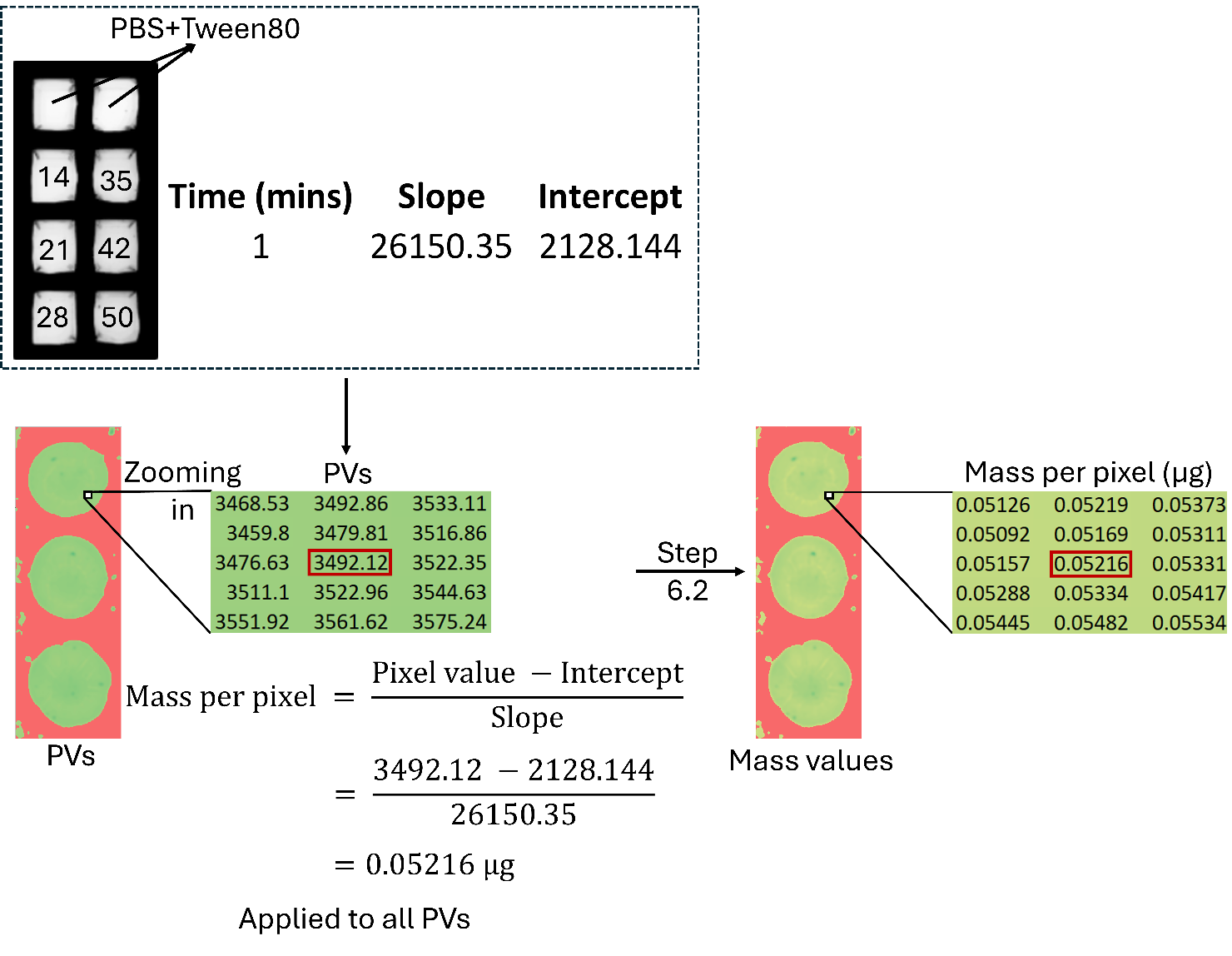


To determine the HA fluorescence, steps 1 to 6.1 are repeated using a blank HA matrix and HA standards. Steps 6.2 to 7 are then used to calculate the mass contributed by HA fluorescence. Starting from step 8, the image processing continues with the b-IgG diffusion images.

HA fluorescence detection:


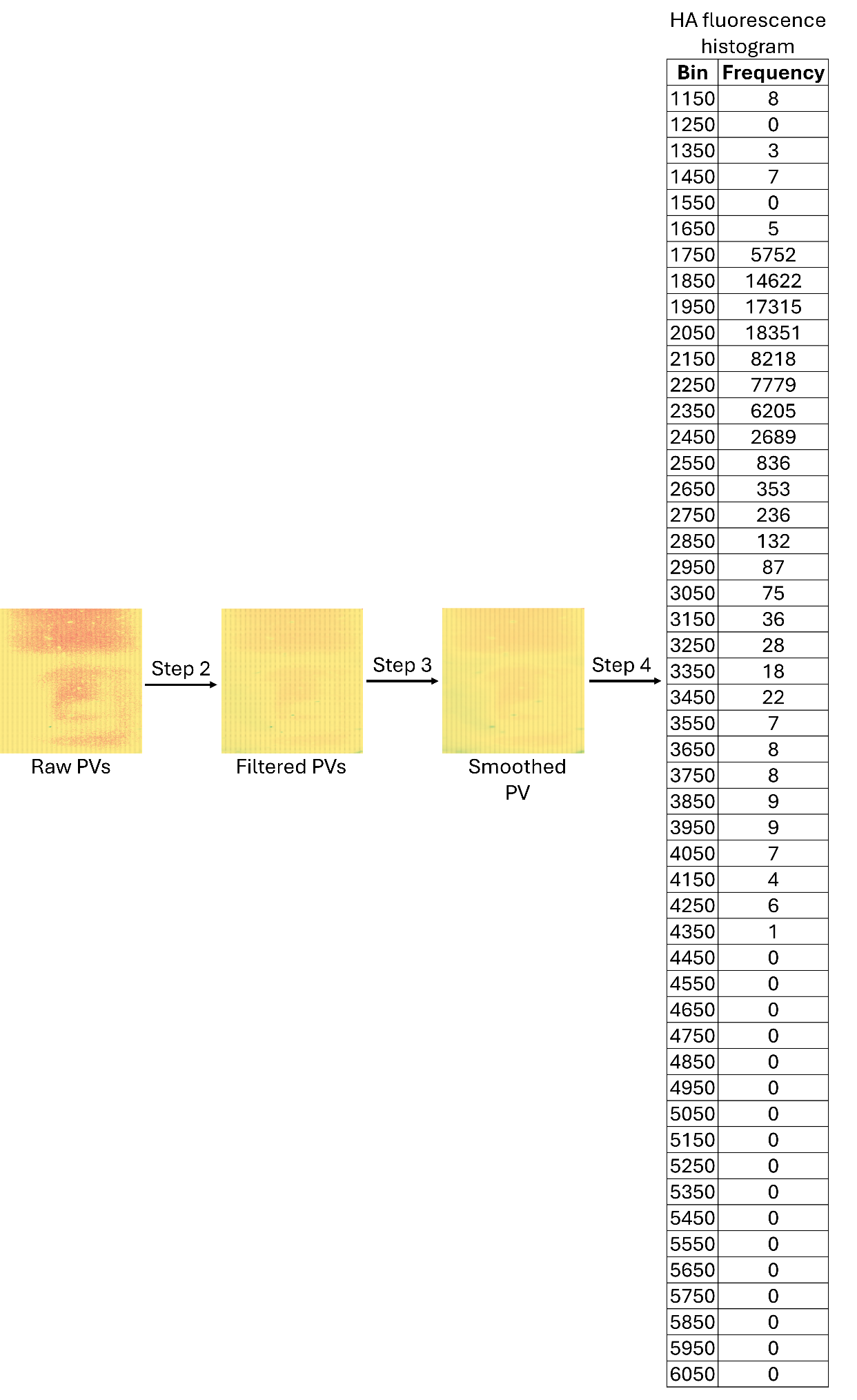


HA fluorescence removal:


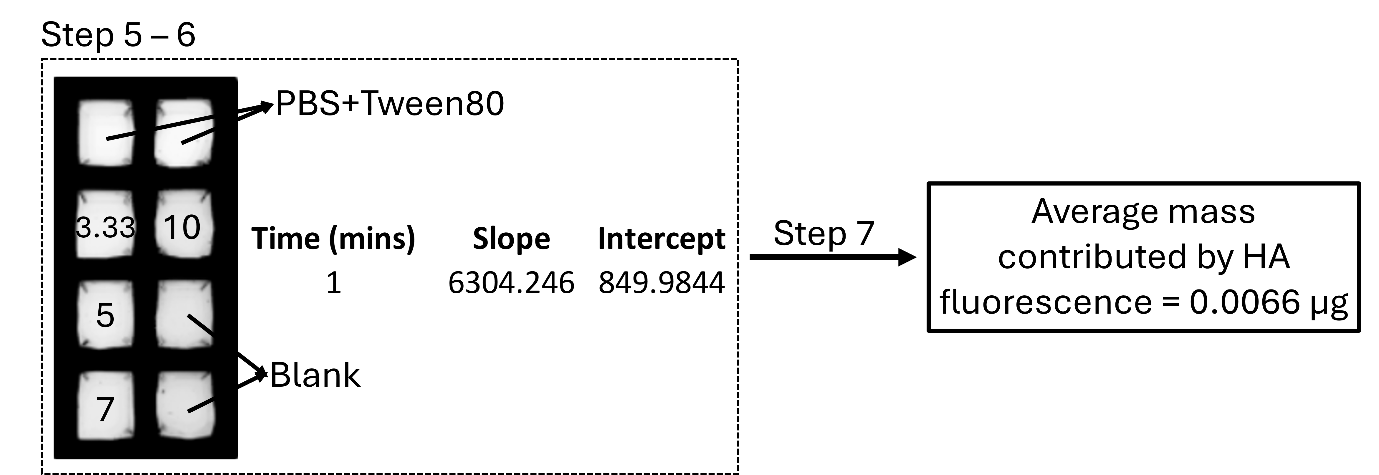


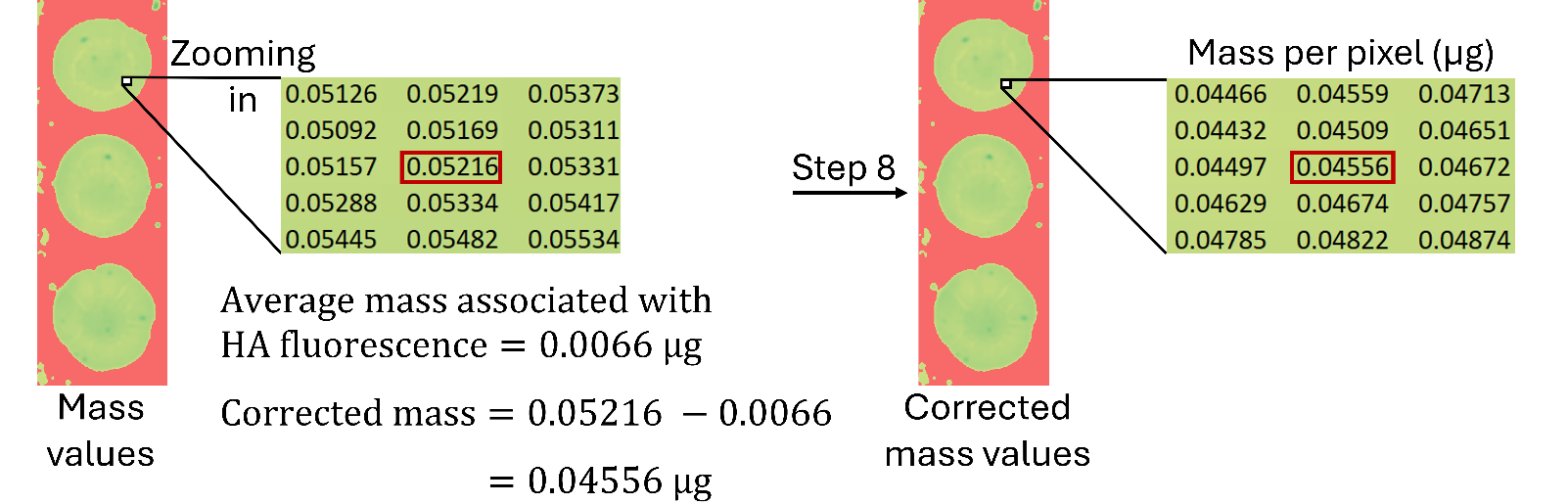


Mass to concentration conversion:


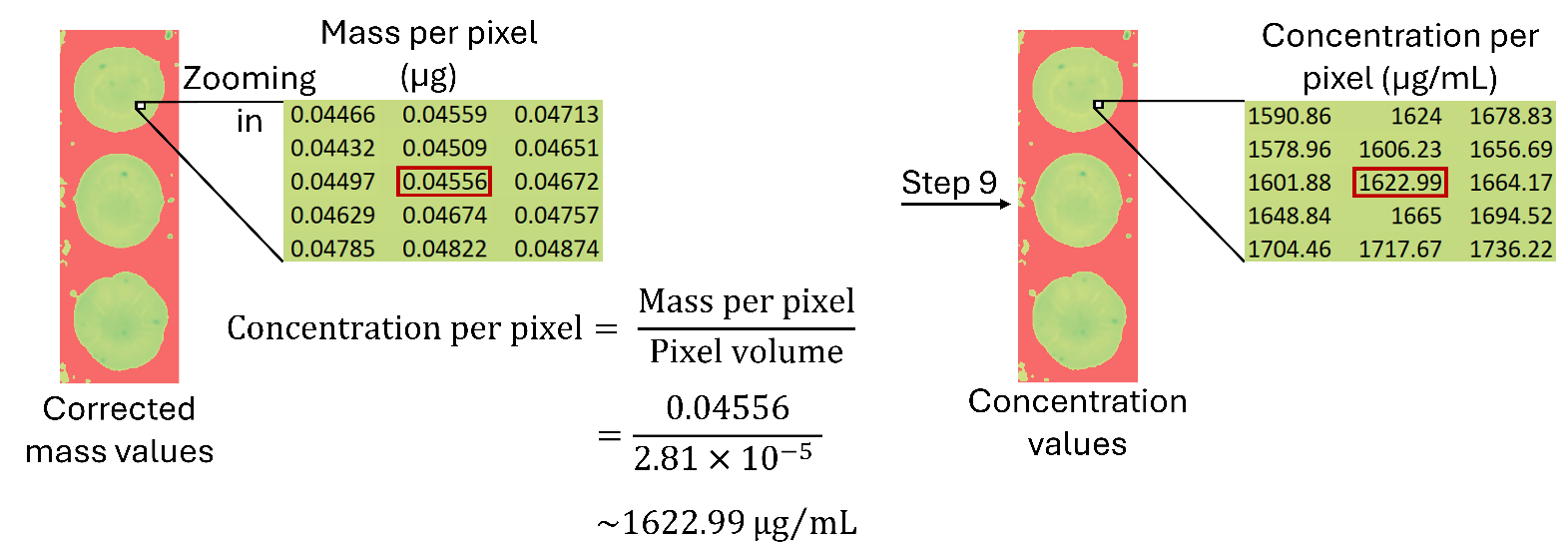


Effective diffusion coefficient (D_eff_) calculation:


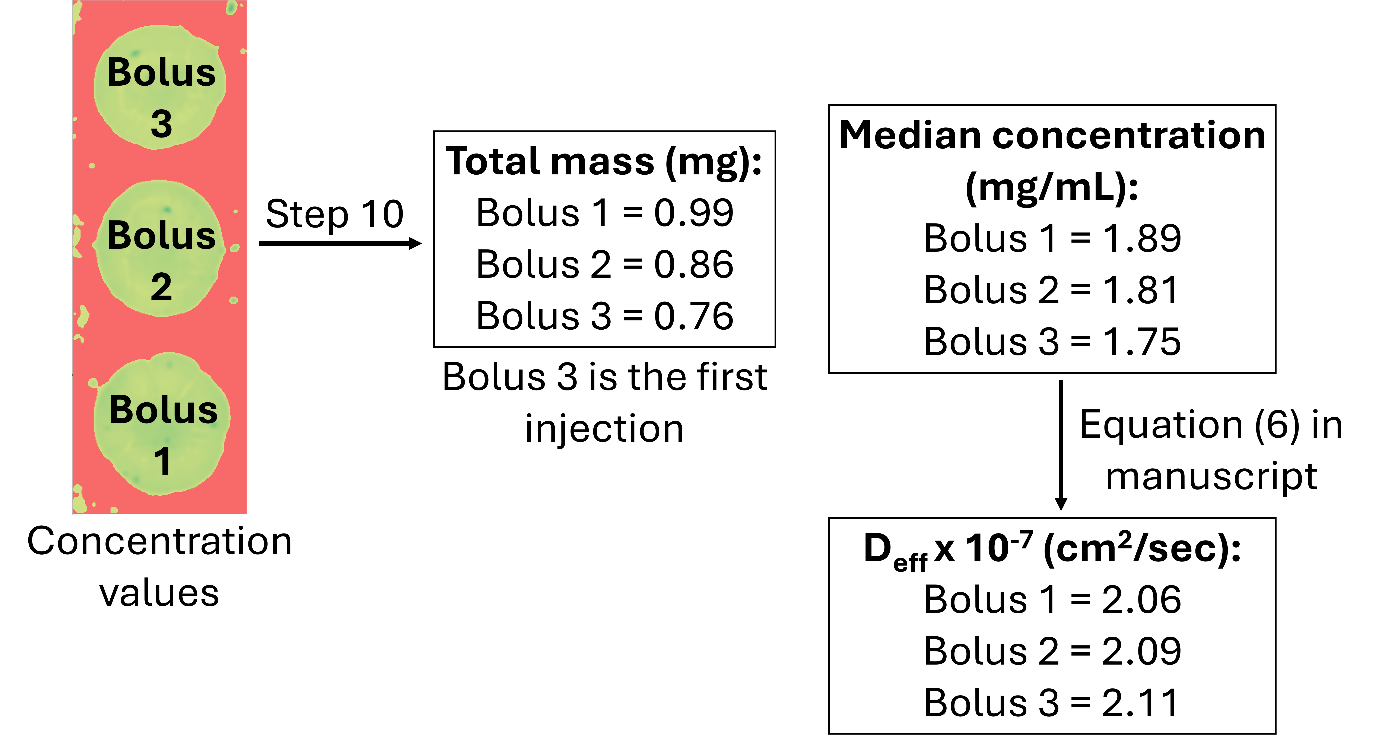

Supplement: Supplementary file 1 — Data S1: Supplementary Information. [file BTPR-42-e70085-s002.docx]
